# Yiqi Yangyin Huoxue Method in Treating IdiopathicPulmonary Fibrosis: A Systematic Review and Meta-Analysis of Randomized Controlled Trials

**DOI:** 10.1155/2020/8391854

**Published:** 2020-10-03

**Authors:** Min Zhou, Chao Ye, Qijun Liang, Qiulan Pei, Fan Xu, Hang Wen

**Affiliations:** ^1^College of Clinical Medical, Jiangxi University of Traditional Chinese Medicine, Nanchang, China; ^2^Affiliated Hospital of Jiangxi University of Traditional Chinese Medicine, Nanchang, China; ^3^College of Clinical Medical, Shanghai University of Traditional Chinese Medicine, Shanghai, China

## Abstract

**Objective:**

Idiopathic pulmonary fibrosis (IPF) is a common respiratory disease that can lead to respiratory failure in severe condition. Despite notable advances in its treatment, some patients show poor effect when treated with conventional western medicine (CWM). Traditional Chinese medicine with the Yiqi Yangyin Huoxue method (YQYYHXM) has been reported to be positive for IPF. In order to explore the effectiveness and safety of YQYYHXM in the treatment of IPF, we performed this meta-analysis.

**Method:**

We searched six databases including Embase, Cochrane, PubMed, CNKI, Wan Fang, and VIP database from their inception to June 1, 2019, and then selected eight studies. Two reviewers independently conducted methodological evaluation and data analysis by the software RevMan 5.3.3 and Stata 12.0.

**Results:**

The meta-analysis revealed that when YQYYHXM was adopted in combination with CWM, cough, chest pain, and shortness of breath of IPF patients improved significantly. After treatment with YQYYHXM combined with CWM, the SGRQ of IPF patients substantially enhanced. YQYYHXM also has positive effect on 6MWD and TLC, but the improvement on FVC was not obvious. In addition, YQYYHXM has no significance in improving PaO_2_. All the adverse events were reported in the control group.

**Conclusion:**

YQYYHXM is more effective and safe as adjunctive treatment for patients with IPF. However, in the future, long-term, large-scale, and high-quality trials will be required to provide more convincing evidence of YQYYHXM due to some limitations of this review.

## 1. Introduction

Idiopathic pulmonary fibrosis (IPF) is an interstitial lung disease (ILD) limited to the lung associated with a severe prognosis (mean survival less than 5 years) and a radiological and histopathological pattern of usual interstitial pneumonia (UIP) [1, 2].The aetiology of IPF is still unknown, but several studies demonstrated a pathogenetic role for epithelial damage, abnormal senescence, and oxidative stress in IPF [3–6]. Especially, increased oxidative stress might promote disease progression in IPF patients mainly in those who are current and former smokers [7]. Epidemiological data showed that its incidence is 4.6 to 8.65 per 100,000, and 6000 people are diagnosed annually. It seems to affect males more than females [8–10]. The poor prognosis of IPF is close to the chronically progressive nature of the disease, resulting in an irreversible damage of lung volume and diffusion capacity that leads to respiratory failure development and death. No therapy can modify IPF natural history (with the only exception for lung transplantation), and actually, the objective of the treatment is to stabilise or reduce the disease progression [11]. Pirfenidone and nintedanib are the only antifibrotic drugs approved by the FDA for reducing functional decline, but they are not enough to halt progression of disease [12, 13].

With the discovery of multiple coactivated pathways applied in the pathogenesis of IPF, single targeted therapy is unlikely to work well in isolation. To date, combination therapy becomes more and more attractive to apply for diagnostic uncertainty and to suppress both profibrotic and proinflammatory pathways at the same time.

Now, there is more and more focus on traditional Chinese medicine (TCM) which is applied for the treatment of respiratory disease more than thousands of years in China [14–16]. Comparatively, TCM is composed of a variety of herb mixtures in one prescription, including a variety of active ingredients, which can work on numerous targets. When combined, they can provide perfect benefit with a moderate effect in a synergistical or antagonistical way [17]. Therefore, TCM has gradually been recognized by the western countries as an complementary treatment drug for IPF and is even widely prescribed as one of the clinical guideline drugs for IPF [18, 19]. Based on the theory of TCM, the diagnosis of IPF was considered as Feiwei, which was firstly mentioned in Huang Di Nei Jing. The symptom characteristic of Feiwei, such as dry cough, progressive dyspnea, tarsalgia, and unfavorable prognosis, was extremely similar to IPF [20, 21].

Based on thousands of years of exploration in clinical experience and summary, the overall pathogenesis of “Feiwei” is deficiency of qi and Yin, and the treatment requires tonifying qi and nourishing Yin. Later, as the research of TCM continues to develop, the famous expert Tianshi Ye put forward the Luo disease theory [22, 23]. Some new treatments of IPF for supplementing qi, nourishing yin, and promoting blood come into being, but the mechanism is unclear and there is no sufficient evidence to prove it. Therefore, this study summarizes the systematic evaluation of TCM treatments of IPF for tonifying qi, nourishing Yin, and promoting blood, aimed to provide some evidence and reference.

## 2. Methods

### 2.1. Search Strategy

Six databases including Embase, Cochrane, PubMed, CNKI, Wan Fang, and VIP database were searched from their inception to June 1, 2019. If any, we would try to contact the original study authors to consult the information we need. We would perform a manual search to track the references of relevant literature. The following were the search keywords and terms we used:“idiopathic pulmonary fibrosis” OR “pulmonary fibrosis” OR “pulmonary interstitial fibrosis” OR “idiopathic pulmonary interstitial fibrosis” OR “IPF” OR “Feibi” OR “Feiwei” AND “Yiqi Yangyin” OR “Yiqi Huoxue” OR “Yiqi Yangyin Huoxue” OR “tonifying qi and nourishing Yin” OR “supplementing qi and promoting blood” OR “nourishing Yin and promoting blood” OR “Traditional Chinese medicine” OR “Chinese medicinal herb” OR “Chinese herbal medicine” OR “combination of Chinese traditional and western medicine” OR “TCM” OR “CHM” AND “randomized controlled trial” OR “randomized trial” OR “controlled clinical trial” OR “clinical research” OR “randomized trial.”

### 2.2. Inclusion and Exclusion Criteria

#### 2.2.1. Inclusion Criteria

① Studies: clinical randomized controlled trials.
② Participants: the patients diagnosed with IPF according to the clinical diagnostic criteria referred to “Guidelines for the Diagnosis and Treatment of Idiopathic Pulmonary Fibrosis in 2002” [24]. There were no limitations on the patient's gender, race, age, and the course and severity of the disease.
③ Interventions: the experimental group was treated with YQYYHXM (decoction or injection and pill) or combined with CWM (conventional western medicine treatment included steroid, acetylcysteine, antibiotics, or noninvasive ventilator-assisted respiratory therapy). And the control group was treated with CWM alone. There was no limitation on course of treatment.
④ Outcomes: the primary outcomess included the changes of TCM symptoms (including changes of cough, changes of chest pain, and changes in shortness of breath) according to Guiding Principles of Clinical Research on New Chinese Medicine in 2002 [25].

The secondary outcomes were forced vital capacity (FVC) and total lung capacity(TLC); partial pressure of oxygen in blood (PaO2); 6-minute walk test distance (6MWT), St. George's Respiratory Questionnaire scores related to health-related quality of life (SGRQ score), and adverse events.

#### 2.2.2. Exclusion Criteria

1. Duplicate literatures.
2. Animal experiments, mechanism research, experience, protocols, and case reports.
3. Literatures on other TCM therapies, for example, acupuncture, asiontophoresis, massage, and acupoint application.
4. Low-quality literature.

### 2.3. Data Abstraction

Based on the inclusion and exclusion criteria, information was filtered and extracted from all included literatures after a comprehensive search by two independent researchers (Min Zhou and Fan Xu). If any, we would consult the third researcher (Qijun Liang) with specific details to resolve discrepancies to make sure to exclude all errors. The selected data included the first author, year of publication, baseline characteristics, intervention methods, duration, outcomes, and classification of disease severity referred to the scoring system of IPF [26] (˃92 = severe; <91 and >66 = moderate; ≤65 = light).

### 2.4. Quality Assessment

The assessment was performed by RevMan 5.3.3 according to the cochrane handbook [27]. The overall assessment was based on details including random sequence generation, allocation hiding, incomplete data, selective reporting, and other bias. If the corresponding details were adequately provided, the risk of bias would be graded to be “low”; otherwise, the risk of bias would be graded to be “high.” If the relevant entry was not presented, it would be assessed to be “unclear.”

### 2.5. Statistical Analysis

We performed the meta-analyses by RevMan 5.3.3 and Stata 12.0 software. In this meta-analysis, we would apply the odds ratio (OR) and the mean difference (MD) to assess dichotomous and continuous variable outcomes with a 95% confidence interval (CI). If *P* < 0.05, it was considered statistically significant. We would use the heterogeneity to evaluate the effect, there was no heterogeneity (*P* > 0.1 or *I*_2_<50%), and we would adopt the fixed effect model for the analysis; otherwise, we would use the random-effects model. We would adopt sensitivity analysis to evaluate the impact of the inclusion study on outcome. And Egger's test was conducted to assess potential publication bias. If *P* < 0.05, this was considered to be of statistical significance. Finally, we adopted the GRADE approach to evaluate the degree of the evidence so as to make our results more convincing.

### 2.6. Quality of Evidence

In this study, we would adopt the GRADE approach to evaluate the degree of the evidence in order to make our results more evidence-based and credible.

## 3. Results

### 3.1. Selection of Study

**Figure 1:**
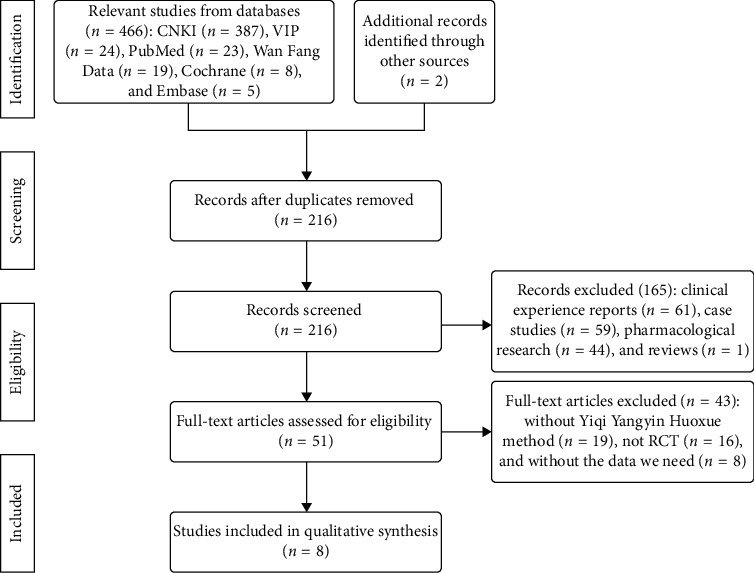
Flow diagram of the study.

A total of 468 potential articles were collected from their inception to June 1, 2019, with extensive search and collection. After duplicates removed by EndNote X7 software, 216 literatures remained. We excluded 165 literatures with comprehensive reasons by screening the title and abstract. 51 literatures remained. After browsing the full-text articles, we excluded 43 articles for the following reasons: without YQYYHXM, not RCT, and without the data we need. Finally, 8 studies [28–35] were collected in this review (Figure 1).

### 3.2. Characteristics of the Eligible Studies

The characteristics of all included studies are summarized in Table 1. All the studies were carried out in China. The sample sizes of these trials ranged from 30 to 120. Treatment duration was from 2 to 12 weeks. The average age was between 55 and 70. The details of illness degree showed that most of the patients had a moderate degree of illness. The experimental group of all the studies was YQYYHXM combined with CWM, only 6 of which were treated with YQYYHXM in the form of decoction, 1 of which was performed with YQYYHXM in the form of injection, and 1 of which was conducted with YQYYHXM in the form of pill. The control group was treated with CWM such as steroid or acetylcysteine, 4 studies of the control group were treated with steroid therapy, and 4 were treated with acetylcysteine.

### 3.3. Description of YQYYHXM

Table 2 describes the specific medicine of the prescriptions. It demonstrates the characteristics of the 8 different formulas researched in the total 8 trials. The partial high frequency Chinese herbs are shown in Table 3. These high-frequency drugs can be classified into three categories according to their characteristic: the herbs of nourishing qi: astragalus, Radix Pseudostellariae, ginseng, and American ginseng; the medicine of nourishing Yin: *Adenophora stricta* Miq, Radix Ophiopogonis, Radix Rehmanniae Praeparata, and Radix Scrophulariae; the medicine of activating blood: ligustrazine, Clematis root, Rhizoma Sparganii, Zedoary, *Salviae miltiorrhiza*, Peach kernel, Safflower, and so on. The partial high frequency herbs are Huangqi (n = 5; rate = 63%), Weilingxian (n = 4; rate = 50%), Maidong (n = 3; rate = 38%), Danshen (n = 3; rate = 38%), Sanleng (n = 3; rate = 38%), and Ezhu (n = 3; rate = 38%). There is a summary of common medicines in Table 3, which is a partial representation of tonifying qi, nourishing yin, and invigorating blood, respectively.

### 3.4. Quality Assessment

**Figure 2:**
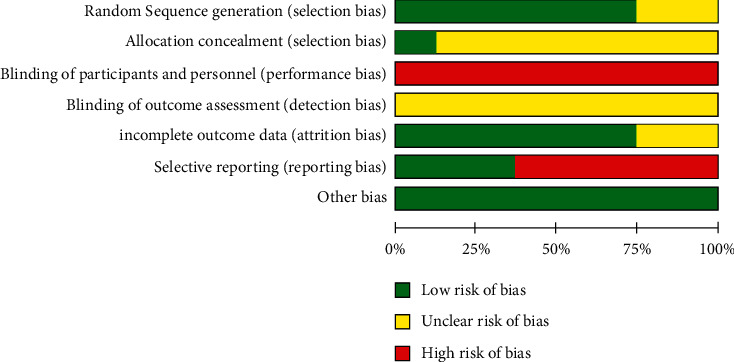
Risk of bias graph.

**Figure 3:**
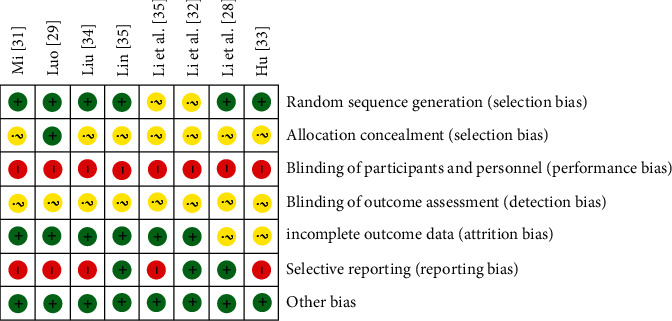
Risk of bias summary.

The risk of bias (ROB) was conducted according to the Cochrane criteria. Two studies [30, 32] only mentioned randomization, but did not elaborate on specific randomization methods which were assessed as “unclear.” Six studies [28, 29, 31, 33–35] reported the numerical randomization method with a low risk of bias; of those, one study [28] performed numerical randomization using SAS.6.12 statistical software. Only one study [29] mentioned allocation concealment which was graded as “low”; others did not report the allocation concealment, and they were evaluated as “unclear.” Additionally, none of the studies involved double blindness in a “high” risk of bias. Four studies [29, 30, 32, 35] described the absence of data during the follow-up; because the number was small, the risk of bias was low. Dropouts were described in five studies [30–32, 34, 35] with the details including quantity and reason in a low risk, which partly was blamed for poor compliance. Three studies [28, 32, 35] reported adverse reactions and described them in detail. All the trials did not involve any other bias in a low risk (Figures 2 and 3).

### 3.5. Evaluation of Meta-Analysis

#### 3.5.1. The Primary Outcomes

**Figure 4:**
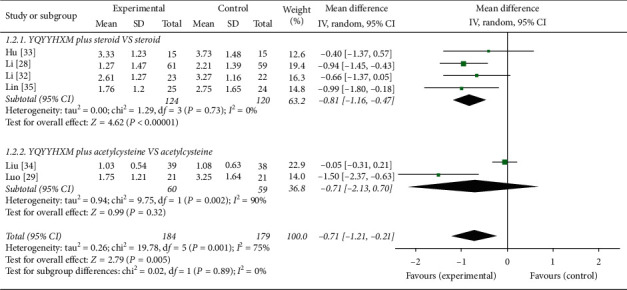
Meta-analysis for the change of cough of YQYYHXM group versus CWM group.

*(1) Change of Cough*. The mean difference (MD) was applied for the analyses according to the 6 included RCTs [28, 29, 32–35]. Data extracted showed that the heterogeneity among the RCTs was obvious (chi-square = 19.78, *P*=0.001, *I*^2^ = 75%), and the random-effects model was used. And the data analysis results illuminated that the YQYYHXM was superior than CWM (MD = −0.71, 95% CI = [−1.21, 0.21], *P*=0.005). The aggregated analysis revealed that there was statistical significance of YQYYHXM combined with steroid compared with steroid alone (chi-square = 1.29, *I*^2^ = 0%, MD = −0.81, 95% CI = [−1.16, 0.47], *P* < 0.00001).However, it was of no statistical difference for YQYYHXM combined with acetylcysteine compared with acetylcysteine alone (*P*=0.32) (Figure 4).

**Figure 5:**
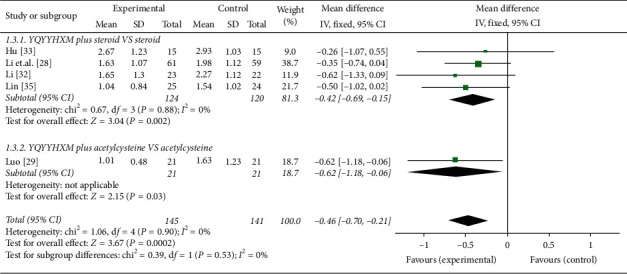
Meta-analysis for the change of chest pain of YQYYHXM group versus CWM group.

*(2) Changes of Chest Pain*. 5 literatures [28, 29, 32, 33, 35] reported the changes of chest pain of SGRQ. And our pooled results showed that the heterogeneity among the RCTs was not found (chi-square = 1.06, *P*=0.90, *I*^2^ = 0%), and the fixed-effects model illuminated that the YQYYHXM was superior than CWM (MD = −0.46, 95% CI = [−0.70, 0.21], *P*=0.0002). Further subgroup analysis revealed that there was statistically significance of YQYYHXM combined with steroid compared with steroid alone (chi-square = 0.67, *I*^2^ = 0%, MD = −0.42, 95% CI = [−0.69,−0.15], *P*=0.002) and acetylcysteine (MD = -0.62, 95% CI = [−1.18, −0.06], *P*=0.03) (Figure 5).

**Figure 6:**
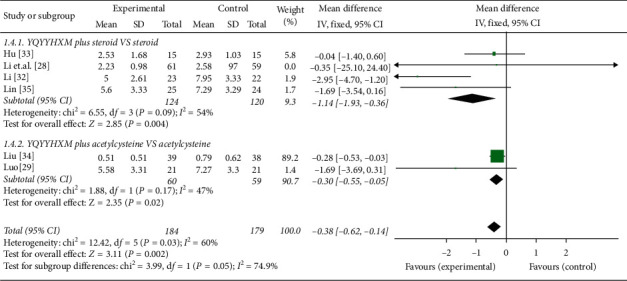
Meta-analysis for the changes in shortness of breath of YQYYHXM group versus CWM group.

*(3) Changes in Shortness of Breath*. 6 literatures [28, 29, 31–33, 35] conducted data analysis of SGRQ score. Findings suggest that the heterogeneity among the RCTs was obvious (chi-square = 12.42, *P*=0.03, *I*^2^ = 60%), and the random-effects model was used. And the data analysis results illuminated that the YQYYHXM combined CWM was superior than CWM alone (MD = −0.38, 95% CI = [-0.62,-0.14], *P*=0.002). The aggregated analysis revealed that there was statistical significance of YQYYHXM combined with steroid compared with steroid alone (chi-square = 6.55, *I*^2^ = 54%, MD = −1.14, 95% CI = [−1.93, −0.36], *P*=0.004) and acetylcysteine alone (chi-square = 1.88, *I*^2^ = 47%, MD = −0.30, 95% CI = [−0.55, −0.05], *P*=0.02) (Figure 6).

### 3.6. The Secondary Outcomes

#### 3.6.1. FVC

**Figure 7:**
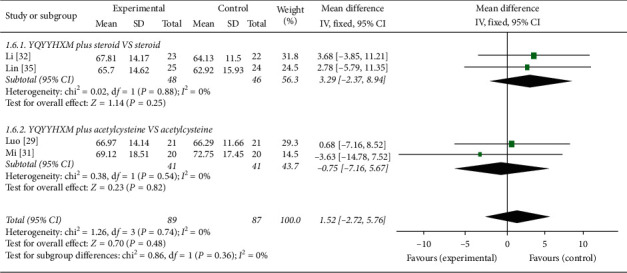
Meta-analysis for FVC of YQYYHXM group versus CWM group.

Four studies [29, 31, 32, 35] provided the data of FVC. Intuitive data showed that the heterogeneity among the RCTs was obvious (chi-square = 1.26, *P*=0.74, *I*^2^ = 0%), and the random-effects model was adopted. The data analysis results illuminated that YQYYHXM combined CWM had no advantage compared with CWM alone (MD = 1.52, 95% CI = [−2.72, 5.76], *P*=0.48). Further subgroup analysis revealed that there was no statistical significance of YQYYHXM combined with steroid (*P*=0.25) and acetylcysteine (*P*=0.82) (Figure 7).

#### 3.6.2. TLC

**Figure 8:**
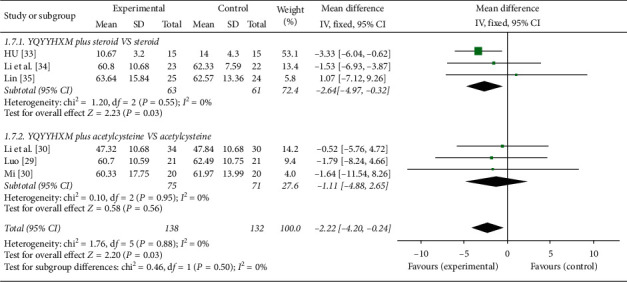
Meta-analysis for TLC of YQYYHXM group versus CWM group.

Five studies [29–33, 35] reported the TLC. Our pooled results suggested that the heterogeneity among the RCTs was not found (chi-square = 1.76, *P*=0.88, *I*^2^ = 0%), and the fixed-effects model was adopted. The aggregated analysis illuminated that YQYYHXM combined CWM was superior than CWM alone (MD = −2.22, 95% CI = [−4.20, −0.24], *P*=0.03).The subgroup analysis revealed that the combination of YQYYHXM and steroid was proved to be more efficient in enhancing TLC than steroid alone (chi-square = 1.20, *I*^2^ = 0%, MD = −2.64, 95% CI = [−4.97, −0.32], *P*=0.03). However, there was no statistically significant difference between YQYYHXM combined with acetylcysteine and acetylcysteine alone (*P*=0.56) (Figure 8).

#### 3.6.3. PaO_2_

**Figure 9:**
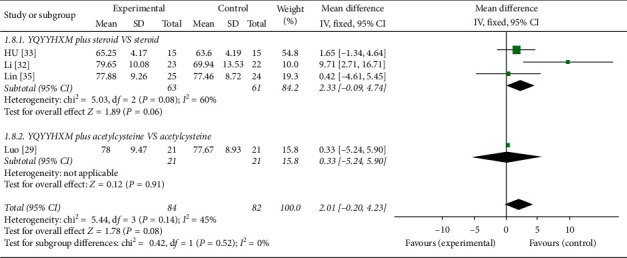
Meta-analysis for PaO_2_ of YQYYHXM group versus CWM group.

Four literatures [29, 32, 33, 35] performed the data analysis of 6MWD. The heterogeneity among the RCTs was not found (chi-square = 5.44, *P*=0.14, *I*^2^ = 45%), and the fixed-effects model suggested that YQYYHXM combined CWM had no statistical significance compared with CWM alone (*P*=0.08). Further subgroup analysis revealed that there was no statistical difference in YQYYHXM combined with steroid (*P*=0.06) and acetylcysteine (*P*=0.91) (Figure 9).

#### 3.6.4. 6MWD

**Figure 10:**
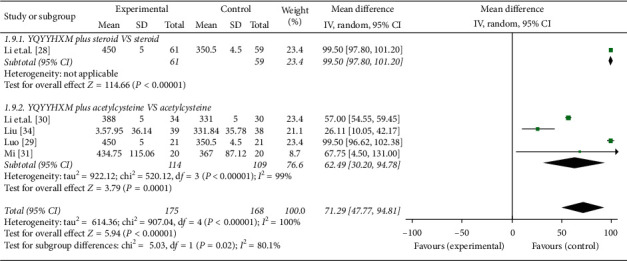
Meta-analysis for 6MWD of YQYYHXM group versus CWM group.

Five literatures [28–31, 34] carried out the data analysis of 6MWD. The heterogeneity among the RCTs was obvious (chi-square = 907.04, *P* < 0.00001, *I*^2^ = 100%), and the random-effects model was used. The data analysis results illuminated that YQYYHXM combined CWM was better at improving the 6MWD than CWM alone (MD = 71.29, 95% CI = [47.77, 94.81], *P* < 0.00001). Further subgroup analysis revealed that there was statistical significance of YQYYHXM combined with steroid (*P* < 0.00001) and acetylcysteine (chi-square = 520.12, *P* < 0.00001, *I*^2^ = 99%, MD = 62.49, 95% CI = [30.20, 94.78], *P* < 0.0001) (Figure 10).

#### 3.6.5. SGRQ Score

**Figure 11:**
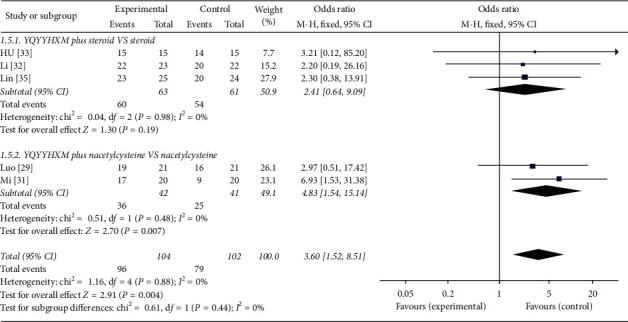
Meta-analysis for SGRQ score of YQYYHXM group versus CWM group.

Five literatures [29, 31, 33, 35] conducted data analysis of SGRQ score. It was obvious that there was no statistical heterogeneity among these studies (chi-square = 1.16, *I*^2^ = 0%). So, the fixed-effects model was applied. Intuitive data showed that, compared with the western medicine group, the TCM group was 3.60 times higher than that in the control group to improve the SGRQ score in the treatment of IPF (OR = 3.60, 95% CI = [1.52, 8.51], *P*=0.04). Further subgroup analysis revealed that there was no statistical significance for YQYYHXM combined with hormone (*P*=0.19). However, it was statistically significant for YQYYHXM combined with acetylcysteine compared with acetylcysteine alone (chi-square = 0.51, *I*^2^ = 0%, OR = 4.83, 95% CI = [1.54, 15.14], *P*=0.007) (Figure 11). Moreover, the quality of evidence was moderate based on the GRADE approach in the statement of facts table for above outcomes. As shown in the table, the quality of change of cough, changes in shortness of breath, PO_2_, 6MWD were moderate, and the rest were low or very low (Table 4).

#### 3.6.6. Adverse Events

**Figure 12:**
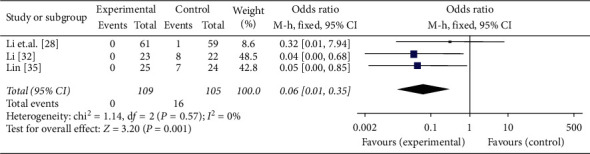
Meta-analysis for adverse events of YQYYHXM group versus CWM group.

Three literatures [29, 31, 33, 35] reported the adverse events. Three studies of YQYYHXM combined with steroid compared with steroid alone [28, 32, 35] reported the details of adverse events. According to the analysis results, the heterogeneity was not found among the three studies (chi-square = 1.14, *P*=0.57, *I*^2^ = 0%). So, the fixed-effects model was applied. Intuitive data showed that there were nineteen adverse events faced by patients who used steroid. And the results expressed that the probability of adverse events about YQYYHXM combined with steroid was more safer than steroid alone in the treatment of IPF (OR = 0.06, 95% CI = [0.01, 0.35], *P*=0.001) (Figure 12).

### 3.7. Evaluation of Heterogeneity and Sensitivity Analysis

**Figure 13:**
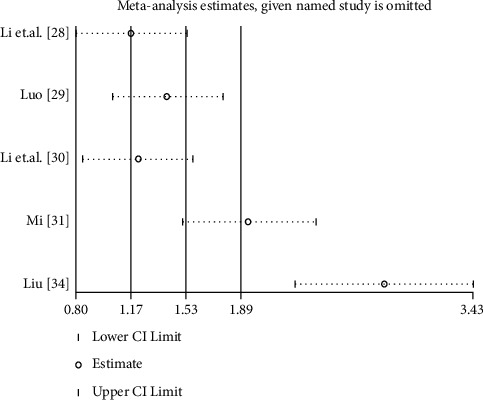
The sensitivity analysis of 6MWD.

Considering obvious heterogeneity in the comparison between YQYYHXM combined with CWM and CWM alone on the 6MWD, change of cough, and shortness of breath, we performed sensitivity analysis. Sensitivity analyses indicated that the result of cough and shortness of breath was stable. However, the results of only one study [34] were heterogeneous with other studies in 6MWD; although this study [34] was excluded, the results of high heterogeneity remained unchanged. Heterogeneity has not been solved, which may be the reason for the form of TCM or more rigorous compatibility (Figure 13).

### 3.8. Publication Bias

Due to the insufficient number of included studies (no more than 10 studies), we analyzed publication bias through Egger's test, and the results showed that there was no significant difference in cough, chest pain, shortness of breath, FVC, TLC, PO_2_, and SGRQ (*P* > 0.05). Nevertheless, there was statistical significance in the 6MWD (*P*=0.013 < 0.05). In the process of extensive search of literature, it was found that almost all the published literatures reported positive results and almost no negative results. And all literatures were published in Chinese. The sources of publication bias may be due to the unpublished negative results or grey literature not identified in this review.

## 4. Pharmacological Research

Nowadays, it is noteworthy to mention that there are more and more specific ingredients of the single herb of TCM which have been discovered to be related to the antipulmonary fibrosis. Maybe TCM is not just a simple compatibility, but the reason for its great effect is closely related to the many pharmacological ingredients it contains.

*Astragalus membranaceus* is one of the representative medicines of one of the representative herbs of tonifying qi. The study [36, 37] demonstrated that astragalus polysaccharides could reduce the expression of matrix metalloproteinase (MMP-2) and matrix metalloproteinase inhibitor (TIMP-1) in the lung tissue of rats with pulmonary fibrosis, thereby inhibiting the process of fibrosis. In vitro experiment found that the damage of lung ultrastructure with pulmonary fibrosis was notably reduced [38]. It significantly suppressed the abnormal increase of interleukin-4 (IL-4) and TNF-*α* levels, increased the level of interferon-*γ* (IFN-*γ*), and contributed to the antifibrotic effects on pulmonary fibrosis by inhibiting TGF-*β*1 production in the lung [39]. In vivo study probed the degrading effects of astragalin on epithelial to mesenchymal transition sensitized by ovalbumin, and the results revealed that astragalin can effectively alleviate bronchial fibrosis by suppressing autophagosome formation in airways [40].

*Salvia miltiorrhiza* Bge, one of the representative herbs of activating blood, Tanshinone IIA, has been shown to reduce TGF-*β*1-induced fibrosis in rat fibroblasts and reduce BLM-induced pulmonary fibrosis. And one of the mechanisms may be involved with regulating pulmonary fibrosis related to inflammatory genes [41]. Lu et al. [42] found that *Salvia miltiorrhiza* was favorable for inhibiting abnormal increase of MDA and HYP and decrease of glutathione (GSH) in lung tissue homogenate of rats with pulmonary fibrosis, so salvia injection liquid has a positive effect of preventing pulmonary fibrosis in rats, but has no reversal effect on formed pulmonary fibrosis. Lin et al. [43] treated pulmonary fibrosis mice with salvia total phenolic acid which significantly reduced pulmonary fibrosis and alveolitis, and HYP levels were also obviously reduced. The in vivo research [44] demonstrated that Tanshinone IIA is beneficial for reducing BLM-induced pulmonary inflammatory cell infiltration, release of proinflammatory cytokine, and excessive collagen deposition in rats.

*Adenophora stricta* Miq. is one of the representative herbs of enriching yin. The relevant experiment confirmed that the work on imbalance of Th1/Th2 with Shashen Maidong decoction to alleviate inflammation and enhance the immune function [45, 46].

It is noteworthy to emphasize that the Feixiantong Decoction was applied in three studies accounting for 38 percent. Through clinical observation and animal experiments, the Feixiantong decoction composed of TCM with YQYYHXM could increase the IFN-*γ* level in the serum and reduce the IL-4 level aimed to regulate the imbalance of Th1/Th2 ratio and reduce the expression of Iand III type collagen fibers, thereby inhibiting ECM overexpression and delaying the process of fibrosis [29, 31, 35]. Related studies have also achieved considerable results under this theory, such as Shenks [41] was composed of TCM with the YQYYHXM. In vivo and in vitro studies have demonstrated that Shenks inhibits fibrosis by blocking the TGF-*β* pathway and regulates the oxidant/antioxidant balance, whether prevention or treatment.

## 5. Discussion

The meta-analysis revealed that when YQYYHXM was adopted in combination with CWM, cough, chest pain, and shortness of breath of IPF patients improved significantly. After treatment with YQYYHXM combined with CWM, the SGRQ of IPF patients substantially enhanced. YQYYHXM also has positive effect on 6MWD and TLC, but the improvement on FVC was not obvious. In addition, YQYYHXM has no significance in improving PaO_2_. All the adverse events were reported in the control group especially in patients who used steroid. In the CWM group, there were 16 cases of adverse reactions, including 9 cases with moderate gastrointestinal reactions, 5 cases with elevated transaminase, and 2 cases with elevated blood glucose. And YQYYHXM combined with steroid in the experimental group reported no adverse reaction. It is important to highlight that it is not advisable to attribute the cause of adverse reactions to the dosage of the steroid. In fact, the dose of steroid is routine dose. Nevertheless, the security of the contrast is obvious, and whether this is the reason for reducing the adverse reactions of steroid or the cause of no adverse reactions caused by symptomatic treatment of TCM is worthy of attention in future studies.

However, this analysis also has some room for improvement. First, the sample size is a bit small, and most of the studies did not mention allocation concealment and blinding. Second, the specific composition of TCM based on the YQYYHXM method is still slightly different, which may be the reason that affects the difference in treatment effect. Third, considering the inherent characteristics of TCM with slow effect and the severity of IPF, the treatment course of all included studies is less than 3 months. For such a serious disease, the observed course is still short. It is difficult to achieve perfect treatment results. With respect to TCM, it is very common in China for the long-term treatment of severe diseases to achieve perfect treatment under the guidance of the principle of syndrome differentiation and treatment. Last, in the process of IPF treatment, the data analysis demonstrated that the tolerance of steroid is poor, resulting in a poorer treatment effect. Therefore, the tolerance is expected to be enhanced in future clinical work. Given those limitations, there is some enlightenment for us that the course of the IPF clinical study in the future should be extended to half a year or one year as much as possible. In addition, increasing need to explore the compatibility of TCM is also a crucial point for treatment of TCM, which is key for TCM to play the ideal effect. Furthermore, making full use of the advantages of good tolerance of TCM is also significant for fully exploiting the value of TCM in the future. Last, more and more high-quality, large-scale, and long-term trials to provide more convincing evidence for the effectiveness and safety of YQYYHXM in the treatment of IPF are required.

## 6. Conclusions

YQYYHXM is more effective and safe as adjunctive treatment for patients with IPF, and it is necessary for further IPF research on YQYYHXM. In the future, high-quality, large-scale, and long-term trials will be required to provide more credible evidence of YQYYHXM.

## Figures and Tables

**Table 1:** Characteristics of included studies.

Studies (first author, year)	Location	Sample size (male/female)	Age (mean years)	Interventions	Duration (weeks)	Outcomes	Classification of disease (light/middle/severe)
Li et al. [28], 2013	Beijing, China	E:40/21 C:38/21	E:61.9 ± 11.2 C:60.7 ± 11.5	E: Feiwei Chongji C: prednisone	8	(1)(2)(3)(9)	NM
Luo [29], 2010	Beijing, China	E:13/8 C:12/9	E:60.98 ± 9.22 C:65.12 ± 8.61	E: Feixiantongfang C:Acetylcysteine	12	(1)(2)(3)(5)(6)(9)	E:5/23/2 C:4/22/4
Li et al. [30], 2015	Beijing, China	E:18/16 C:14/16	E:58.23 ± 8.35 C:59.98 ± 7.43	E: Yangyin Tongluo pill C: acetylcysteine	12	(5)(9)	NM
Mi [31], 2015	Beijing, China	E:13/7 C:11/9	E:65 ± 9.453 C:68.1 ± 12.161	E: Feixiantongfang C: acetylcysteine	12	(4)(5)(9)	NM
Li et al. [32], 2014	Beijing, China	E:14/9 C:13/9	E:59.97 ± 9.77 C:64.59 ± 10.37	E: Feibitongfang C: prednisone	12	(1)(2)(3)(4)(5)(6)	E:0/18/3 C:0/17/5
Hu [33], 2007	Beijing, China	E:10/5 C:9/6	E:63.9 ± 9.4 C:65.6 ± 8.7	E: Baofeitang and Dahuangzhechong pill C: prednisone	12	(1)(2)(3)(4)(5)(6)	E:3/12/0 C:1/13/1
Liu [34], 2017	Henan, China	E:29/11 C:28/12	E:66. 49 ± 1. 11 C:66. 03 ± 1. 16	E: Shenmai injection + Shenxiong injection + C C: acetylcysteine + do xofylline injection	2	(1)(3)(9)	NM
Lin [35], 2007	Beijing, China	E:15/10 C:14/10	E:61.12 ± 9.22 C:65.04 ± 8.62	E: Feixiantongfang C: prednisone	12	(1)(2)(3)(4)(5)(6)(7)(8)	E:3/18/4 C:2/9/3

(1), change of cough; (2), changes of chest pain; (3), changes in shortness of breath; (4), lung function vital capacity (FVC); (5), total lung capacity (TLC); (6), partial pressure of oxygen in blood (PaO_2_); (7), adverse events; (8), SGRQ score; (9), 6-minute walk test distance (6MWT); NM: not mentioned; E: experimental group; C: control group.

**Table 2:** Description of the prescriptions.

First author	Formula	Ingredient (Latin name)	Chinese name	Dosage (g)
Li et al. [28]	Feiwei Chongji	(1) *Panax quinquefolius* L. (2) *Panax notoginseng* (Burk) F. H. Chen (3) *Cornus officinalis* Sieb. et Zucc (4) *Schisandra chinensis* (5) *Aster tataricus* L.f. (6) *Ophiopogon japonicus* (L.f.) Ker Gawl (7) *Ginkgo biloba* L. (8) *Glycyrrhiza uralensis* Fisch.	(1) Xiyangshen (2) Sanqifen (3) Shanyurou (4) Wuweizi (5) Ziwan (6) Maidong (7) Yinxingye (8) Zhigancao	15 3 15 15 15 15 10 10

Luo [29]	Feixiantong	(1)*Astragalus membranaceus* (2) *Rhodiola crenulata* (Hook. f. et Thoms.) H. Ohba (3) Clematis du sis Osbeck (4) Pumice stone (5) *Sparganium stoloniferum* Buch.-Ham (6) *Inula japonica* Thunb. (7) *Curcuma phaeocaulis* Val. (8) *Rehmannia glutinosa* Libosch (9) *Glycyrrhiza uralensis* Fisch.	(1) Shenghuangqi (2) Hongjingtian (3) Weilingxia (4) Haifushi (5) Sanleng (6) Xuanfuhua (7) Ezhu (8) Shengdihuang (9) Gancao	30 15 12 15 10 15 10 30 6

Li et al. [30]	Yangyin Yife Tongluo pill	(1) *Astragalus membranaceus* (2) *Panax quinquefolius* L (3) *Stemona sessilifolia* (Miq.) Miq. (4) *Ophiopogon japonicus* (L.f.) Ker Gawl (5) *Fritillaria cirrhosa* D.Don (6) *Paeonia lactiflora* Pall (7) *Scrophularia ningpoensis* Hemsl (8) *Citrus reticulate* Blanco (9) *Salvia miltiorrhiza* Bge (10) *Amygdalus persica* (L.) Batsch (11) *Saposhnikovia divaricata* (Turcz.) Schischk (12) *Ligustrum lucidum* Ait. (13) *Gekko gecko* Linnaeus	(1) Huangqi (2) Xiyangshen (3) Baizhu (4) Maidong (5) Chuanbeimu (6) Chishao (7) Xuanshen (8) Juhong (9) Danshen (10) Taoren (11) Fangfeng (12) Nvzhenzi (13) Gejie	6 g per pill

Mi [31]	Feixiantong	(1) *Astragalus membranaceus* (Fisch.) Bunge (2) Carapax Trionycis (3) Clematis du sis Osbeck (4) Pumice stone (5) *Sparganium stoloniferum* Buch.-Ham (6) *Inula japonica* Thunb. (7) *Curcuma phaeocaulis* Val. (8) *Rehmannia glutinosa* Libosch (9) *Glycyrrhiza uralensis* Fisch.	(1) Shenghuangqi (2) Biejia (3) Weilingxian (4) Haifushi (5) Sanleng (6) Xuanfuhua (7) Ezhu (8) Shengdihuang (9) Gancao	30 10 12 15 10 15 10 30 6

Liu et al. [32]	Feibitongfang	(1) Clematis du sis Osbeck (2) *Astragalus membranaceus* (Fisch.) Bunge var. mongholicus (Bunge) P.K.Hsiao (3) *Ligusticum chuanxiong* Hort. (4) *Glehnia littoralis* Fr. Schmidt ex Miq. (5) *Stemona sessilifolia* (Miq.) Miq. (6) *Aster tataricus* L.f (7) *Glycyrrhiza uralensis* Fisch.	(1) Weilingxian (2) Huangqi (3) Chuanxiong (4) Shashen (5) Baibu (6) Ziwan (7) Gancao	15 20 10 20 10 10 10

Hu [33]	Baofeitang and Dahuangzhec hong pill	(1) *Salvia miltiorrhiza* Bge (2) *Ophiopogon japonicus* (L.f) Ker Gawl (3) *Schisandra chinensis* (Turcz.) Baill (4) *Angelica sinensis* (Oliv.) Diels (5) *Fritillaria thunbergii* Miq. (6) *Sophora flavescens* Ait. (7) Spina Gleditsiae (8) *Pinellia ternata* (Thunh.) Breit.	(1) Dangshen (2) Maidong (3)Wuweizi (4) Danggui (5) Zhebei (6) Kushen (7) Zaoci (8) Banxia	NM

Li [34]	SFJ and SXJ	(1) *Panax ginseng* C.A.Mey (2) *Aconitum carmichaelii* Debx. (3) *Salvia miltiorrhiza* Bge (4) *Ligusticum chuanxiong* Hort.	(1) Renshen (2) Fuzhi (3) Danshen (4) Chuanxiong	NM

Lin [35]	Feixiantong	(1) *Astragalus membranaceus* (Fisch.) Bunge var. mongholicus (Bunge) P.K.Hsiao (2) Carapax Trionycis (3) Clematis du sis Osbeck (4)Pumice stone (5) *Sparganium stoloniferum* Buch.-Ham (6) *Inula japonica* Thunb. (7) *Curcuma phaeocaulis* Val. (8) *Rehmannia glutinosa* Libosch (9) *Glycyrrhiza uralensis* Fisch.	(1) Shenghuangqi (2) Biejia (3) Weilingxian (4) Haifushi (5) Sanleng (6) Xuanfuhua (7) Ezhu (8) Shengdihuang (9) Gancao	NM

SFJ: Renshen Fuzhi injection; SXJ: Danshen Chuanxiong injection; NM: not mentioned.

**Table 3:** Frequency statistics of herbs that appeared in eight trials.

Scientific name	Chinese name	Frequency	Rate (%)	TCM diagnosis
*Astragalus membranaceus* (Fisch.) Bunge var.mongholicus (Bunge) P.K.Hsiao	Huangqi	5	63	Qi deficiency
Clematis du sis Osbeck	Weilingxian	4	50	Blood stasis
*Ophiopogon japonicus* (L.f) Ker Gawl	Maidong	3	38	Yin deficiency
*Salvia miltiorrhiza* Bge	Danshen	3	38	Blood stasis
*Sparganium stoloniferum* Buch.-Ham	Sanleng	3	38	Blood stasis
*Curcuma phaeocaulis* Val.	Ezhu	3	38	Blood stasis

**Table 4:** Statement of facts for all outcomes.

YQYYHXM plus CWM vs. CWM					
Patient or population: patients with idiopathic pulmonary fibrosis; setting: all eligible patients with intervention therapy					
Intervention: (YQYYHXM, CWM)					

Outcome	No. of participants (RCTs)	Relative effect (95% CI)	Anticipated absolute effects (95% CI)		Quality of the evidence
			CWM	CWM + YQYYHXM	(GRADE)

Change of cough Follow-up: 2–12 days	363 (6 RCTs)		The mean of change of cough was 1.08 to 3.73	MD 0.71 lower (1.21 to 0.21 lower)	⊕⊕⊕Ο moderate^ab^

Changes of chest pain Follow-up: 8–12 days	286 (5 RCTs)		The mean of chest pain was 1.63 to 2.93	MD 0.46 lower (0.7 to 0.21 lower)	⊕⊕ΟΟ low

Changes in shortness of breath Follow-up:2–12 days	363 (6 RCTs)		The mean of shortness of breath was 0.79 to 7.95	MD 0.38 lower (0.62 to 0.14 lower)	⊕⊕⊕Ο moderate^a^

FVC Follow-up: 12 days	176 (4 RCTs)		The mean of FVC was 62.92 to 72.25	MD 1.52 higher (2.72 lower to 5.76 higher)	⊕⊕ΟΟ low^a^

TLC Follow-up: 12 days	270 (6 RCTs)		The mean of TLC was 47.84 to 62.57	MD 2.22 lower (4.2 to 0.24 lower)	⊕⊕ΟΟ low^ac^

PO_2_ Follow-up: 12 days	166 (4 RCTs)		The mean of PO_2_ was 77.46 to 77.67	MD 2.01 higher (0.2 lower to 4.23 higher)	⊕⊕⊕Ο moderate^a^

6MWD Follow-up: 2–12days	343 (5 RCTs)		The mean of 6MWD was 331 to 350.5	MD 87.96 higher (86.71 to 89.21 higher)	⊕⊕⊕Ο moderate^ac^

SGRQ Follow-up:2–12 days	206 (5 RCTs)	OR 3.6 (1.52 to 8.51)	The mean of SGRQ 114 per 1000 (50 to 144)	151 per 1000 (65 to 192)	⊕ΟΟΟ very low^a^

^*∗*^GRADE Working Group grades of evidence. High quality: we are extremely confident for that the effect estimation lies close to true effect. Moderate quality: we have enough confidence for that effect estimation is likely to be close to the true effect. Low quality: we maintain partial confidence for that effect estimation may be obviously different from true effect. Very low quality: we have little confidence for that effect estimation is likely to be obviously different from true effect. Explanations. _a_No blinding. _b_High heterogeneity. *cP* < 0.05 in Egger's test.
